# Validating a scoring tool to predict acute kidney injury (AKI) following cardiac surgery

**DOI:** 10.1186/s40697-015-0037-x

**Published:** 2015-01-30

**Authors:** Brian Wong, Jennifer St. Onge, Stephen Korkola, Bhanu Prasad

**Affiliations:** College of Medicine – Regina Campus, University of Saskatchewan, Regina General Hospital, 1440-14th Avenue, Regina, SK S4P0W5 Canada; Research and Performance Support, Regina Qu’Appelle Health Region, Regina, SK, 2180 – 23rd Avenue, Regina, SK S4S 0A5 Canada; Department of Surgery, Regina Qu’Appelle Health Region, Regina, SK, Regina General Hospital, 1440-14th Avenue, Regina, SK S4P0W5 Canada; Section of Nephrology, Department of Medicine, Regina Qu’Appelle Health Region, Regina, SK, Regina General Hospital, 1440-14th Avenue, 3401-B Pasqua Street, Regina, SK S4P0W5 Canada

**Keywords:** Dialysis, Mortality, Cardiac surgery complication, Serum creatinine, Acute kidney injury

## Abstract

**Background:**

Acute kidney injury (AKI) after cardiac surgery is associated with an increased risk of mortality. Preoperative risk scores can identify patients at risk for AKI and facilitate preventive strategies. Currently, validated risk scores are used to predict AKI requiring dialysis (AKI-D); less is known about whether these tools predict less severe forms of AKI.

**Objective:**

To evaluate the Cleveland Clinic scoring tool in predicting both AKI-D and less severe stages of AKI in patients after cardiac surgery in a Canadian tertiary care center.

**Design:**

Retrospective case–control study.

**Setting:**

Regina Qu’Appelle Health Region (RQHR) from 2007 to 2011.

**Patients:**

Patients who underwent cardiac surgery and developed postoperative kidney injury (n = 2316).

**Measurements:**

Data on risk factors for AKI and outcomes of cardiac surgery were collected from a retrospective chart review.

**Methods:**

The primary outcome was AKI, defined as Stage 1 (increase in serum creatinine 1.5-1.9 X baseline within 5 days), Stage 2 (increase 2.0-2.9 X baseline), or Stage 3 (increase 3.0 X baseline or more OR initiation of dialysis during hospital stay). We assessed the performance of a modified version of the Cleveland Clinic tool using receiver operating curve analyses.

**Results:**

The incidence of AKI was 6.1% (Stage 1), 2.6% (Stage 2), and 5.8% (Stage 3). The area under the curve (AUC) for the Cleveland score was 0.61 (95% CI: 0.56 to 0.65; *p* < 0.001) for Stage 1, 0.61 (95% CI: 0.54 to 0.68; *p* < 0.01) for Stage 2, and 0.78 (95% CI: 0.74 to 0.82; *p* < 0.001) for Stage 3. Greater level of risk on the Cleveland tool was associated with a higher risk of Stage 3 AKI.

**Limitations:**

Lack of prospective validation.

**Conclusions:**

The modified Cleveland Clinic tool was valid in identifying patients with severe stages of AKI but did not have strong discrimination for early AKI stages.

**Electronic supplementary material:**

The online version of this article (doi:10.1186/s40697-015-0037-x) contains supplementary material, which is available to authorized users.

## Abrégé

**Contexte:**

L’insuffisance rénale aiguë (IRA) survenant après une chirurgie cardiaque est liée à un risque accru de mortalité. Divers outils d’évaluation du risque préopératoire permettent de dépister les patients à risque d’IRA, facilitant ainsi les stratégies de prévention. Présentement, les scores de risque validés sont utilisés pour prédire une IRA nécessitant une dialyse (IRA-D); nous avons toutefois peu d’information sur l’utilité de ces outils d’évaluation pour prédire des formes moins sévères d’IRA.

**Objectif de l’étude:**

Déterminer la valeur prédictive de l’outil d’évaluation de la Cleveland Clinic, en ce qui concerne l’IRA-D et des formes moins sévères d’IRA, chez des patients en phase postopératoire cardiaque, dans un établissement de soins tertiaires canadien.

**Type d’étude:**

Étude cas-témoins.

**Contexte:**

Regina Qu’Appelle Health Region (RQHR) pour la période de 2007 à 2011.

**Patients:**

Échantillon de patients ayant subi une chirurgie cardiaque, et ayant développé une IRA postopératoire (n = 2316).

**Mesures:**

Les données concernant les facteurs de risque d’IRA, et les résultats de la chirurgie cardiaque ont été colligés à partir d’un examen rétrospectif des dossiers.

**Méthode:**

La première complication rapportée était l’IRA : de stade 1 (avec élévation du taux de créatininémie de 1,5 -1,9 X par rapport à la valeur de référence, à l’intérieur de 5 jours); de stade 2 (élévation de 2,0-2,9 X la valeur de référence), ou de stade 3 (élévation minimale de 3 X la valeur de référence ou dialyse initiée pendant le séjour à l’hôpital). Nous avons évalué la performance d’une version modifiée de l’outil d’évaluation de la Cleveland Clinic en analysant la courbe ROC (receiver operating curve).

**Résultats:**

La fréquence d’IRA était de 6,1% (stade 1), 2,6% (stade 2), et de 5,8% (stade 3). L’aire sous la courbe, pour le score de Cleveland était de 0,61 (IC 95%: de 0,56 à 0,65; p < 0.001) pour l’IRA de stade 1, de 0,61 (IC 95%; de 0,54 à 0,68; p < 0.01) pour le stade 2, et de 0,78 (IC 95%: de 0,74 à 0,82; p < 0.001) pour le stade 3. Un risque accru avec l’outil d’évaluation de la Cleveland Clinic était lié à un risque accru d’IRA de stade 3.

**Limites de l’étude:**

Absence de validation prospective.

**Conclusions:**

La version modifiée de l’outil d’évaluation de la Cleveland Clinic dépistait adéquatement les patients atteints de l’IRA dans ses formes sévères, mais ne permet aucune distinction claire pour ce qui est des stades moins sévères de l’IRA.

## What was known before

The Cleveland Clinic score has consistently had the highest discrimination when compared to other risk scores that predict AKI requiring dialysis (AKI-D), and it has been validated in multiple cohorts for AKI-D. However, it has not been validated in a Canadian cohort for less severe forms of AKI.

## What this adds

We demonstrated in a Canadian cohort that the Cleveland Clinic score was valid in predicting severe AKI (Stage 3), but less successful in predicting patients with less severe forms of AKI (Stage 1 and Stage 2).

## Background

Acute kidney injury (AKI) after cardiac surgery is a serious complication due to its association with elevated mortality [1-5]. Depending on the definition, up to 30% of cardiac surgery patients develop some form of AKI post-surgery [2,5-11], and 1-5% of patients develop the most severe form of kidney injury: AKI requiring dialysis (AKI-D) [2,8,11,12]. Mortality following AKI-D has been reported to be greater than 50%, and in some cases, as high as 80% [1,10,13-15]. In contrast, mortality associated with cardiac surgery alone ranges from 2 to 8% [8,16,17].

Even mild forms of AKI can impact short- and long-term morbidity and mortality [7,10,18-21]. Minor (0–44.2 μmol/L) postoperative increases in serum creatinine (SCr) from baseline can increase the risk of 30-day mortality 3-fold [10]; and larger SCr increases (≥44.2 μmol/L) were associated with a >18-fold elevation [10]. Less severe forms of AKI after cardiac surgery are also associated with an increased risk of other adverse outcomes, such as progression of chronic kidney disease (CKD) [22], increased postoperative length of stay [23], and increased risk of 30-day hospital readmission [24]. The short- and long-term outcomes associated with less severe forms of AKI indicate that prevention and treatment measures are desperately needed.

Currently, there is no clear evidence for an effective preventive or therapeutic pharmaceutical agent for AKI [1,3,8,25,26]. Preoperative risk stratification can contribute to informed decision making, increased awareness among multiple care providers, hemodynamic and fluid optimization, and future research studies [27]. There are a few externally valid scoring tools for risk stratification for AKI-D [8]. Using major AKI-D risk factors, Thakar et al. [16] developed the Cleveland Clinic scoring tool in a large cohort of patients (n = 15,838) to identify patients at risk of developing AKI-D after open-heart surgery. Of the available AKI-D scoring tools, multiple comparison studies have found that the Cleveland Clinic score has the highest discriminative power, and it has performed well in some external cohorts [6,27-29]. However, a key limitation of this risk score is that it does not predict milder forms of AKI [14,30,31], which still have significant short- and long-term health effects.

The purpose of this study was to assess the performance of the Cleveland Clinic scoring tool in predicting all stages of AKI in a Canadian tertiary care center providing cardiac surgery.

## Methods

### Data collection and study cohort

We conducted a retrospective chart review of all patients who underwent cardiac surgery from 2007 to 2011 in the RQHR (n = 2343). The data for all variables except serum creatinine were obtained from a database produced by the health records department who previously coded and entered the data from patient hospital charts. Data for serum creatinine was obtained using an electronic repository that houses all lab data in the hospital. We used the Medical Information Quality System (MIQS) to identify patients who required intermittent hemodialysis if there were any discrepancies in the data.

We collected data on the following variables that were predictors of AKI in the Cleveland Clinic scoring tool [16]: gender; comorbidities including congestive heart failure (CHF), type 1 diabetes, and chronic obstructive pulmonary disease (COPD); cardiac indicators including left ventricular ejection fraction (LVEF) <35%, preoperative use of an intra-aortic balloon pump (IABP), and history of previous cardiac surgery; type of current cardiac surgery; and preoperative creatinine (μmol/L). We were not able to obtain data on insulin use in patients with diabetes; therefore we focused only on type 1 diabetics. We also did not have information on medication use in COPD so all patients with this diagnosis were included. The types of cardiac surgery included coronary artery bypass graft (CABG), aortic valve repair or replacement (AVR), mitral valve repair or replacement (MVR), tricuspid valve repair or replacement (TVR), and combinations of CABG and AVR, MVR and TVR, as well as other cardiac surgeries such as ventricular aneurysm repair, pericardiectomy, etc. Emergent surgery data was not used because it was not consistently or accurately recorded in the patients' charts.

In addition, we collected data on other preoperative variables (age, BMI), perioperative variables [operating room time (open to close; minutes), operation time (entry to exit; minutes), clamp time (minutes), pump time (minutes), number of bypass grafts], and postoperative variables [length of stay (days) and dialysis modality if required (hemodialysis, continuous, peritoneal dialysis)]. For preoperative creatinine, we used data from the sample that was reported by the lab at the time closest to the start of the surgery (up to and within 5 minutes after the start of surgery). This included lab samples taken up to 3 months prior to the surgery in rare cases. For postoperative creatinine, we collected data from the highest creatinine sample on each postoperative day for five consecutive days, and also the highest sample on the final day of hospital stay if the patient stayed more than five days. If there were no data on a given postoperative day, it was left blank.

### Outcomes

Our primary outcome was AKI Stage 1, Stage 2 or Stage 3. Stage 1 was defined as an increase in serum creatinine 1.5-1.9 X baseline within 5 days. Stage 2 was an increase in serum creatinine 2.0-2.9 X baseline within 5 days, and Stage 3 was an increase in serum creatinine 3.0 X baseline or more OR initiation of dialysis anytime during hospital stay. The groups were mutually exclusive. Due to limitations in data availability up to 7 days and urine output, we were not able to use the formal KDIGO criteria for AKI [32].

### Cleveland clinic score

To evaluate the use of the scoring tool, we calculated a score for each patient based on the general method described previously (Thakar et al. [16]; Additional file 1). As noted above, we were limited to using type 1 diabetes instead of insulin-requiring diabetes, and were unable to include emergency surgery in the scoring tool. Therefore, the minimum score for a patient was 0 and the maximum was 15 (instead of 17).

### Statistical analyses

Descriptive statistics for categorical variables were reported as frequency (percentage), whereas continuous variables were reported as means (standard deviation). Categorical variables were compared between AKI Stages 1–3 using chi-square tests, and continuous variables were compared using one-way ANOVAs. Logistic regression models and receiver operator curve analyses were used to calculate area under the curve (AUC) for the prediction of AKI Stage 1, 2 and 3 using the Cleveland score. All statistical tests were 2-sided, with alpha level set at 0.05 for statistical significance. Statistical analyses were performed using SPSS Version 17.0. The Regina Qu’Appelle Health Region and the University of Saskatchewan Research Ethics Boards through a harmonized review approved the study.

## Results

A total of 2343 patients had cardiac surgery performed between 2007 and 2011. Two patients did not have complete creatinine data, and twenty-five patients were excluded from the data due to a history of requiring dialysis (either hemodialysis or peritoneal) prior to surgery, leaving a final sample of 2316 patients.

The incidence of AKI was 6.1% (n = 142) in Stage 1, 2.6% (n = 60) in Stage 2, and 5.8% (n = 134) in Stage 3. Of the total 134 Stage 3 patients, 125 (5.4% of total) patients required dialysis during their hospital stay. Seventy-six of the 125 patients (60.8%) received in-patient hemodialysis, 45 (36%) received continuous renal replacement therapy, and 1 (1%) patient received peritoneal dialysis; for 4 others (3.2%), the type of dialysis was not recorded.

We compared patients without AKI to patients with AKI Stages on all potential risk factors using chi-square analyses and one-way ANOVA’s as appropriate. Patients in the four groups were similar in terms of BMI, and the number of bypass grafts (Table 1). A history of previous cardiac surgery, preoperative use of an IABP, presence of CHF, type of surgery, as well as intraoperative risk factors such as longer clamp time and pump time were associated with more severe AKI (Table 1). The overall relationship between diabetes and AKI Stage was significant (*p* = 0.01), but this was largely driven by a large percentage of patients in Stage 2 with type 2 diabetes; the p-value for type 1 diabetes alone was not significant (*p* = 0.7). Patients with increased severity of AKI had a significantly longer length of stay in hospital (32 vs. 14 days; Table 2) and were much more likely to die in hospital (47.8% vs. 2.5%).

**Table 1:** **Preoperative and perioperative risk variables associated with KDIGO stages 1-3**

**Risk factors (Total n = 2316)**	**No AKI (N = 1980)**	**KDIGO stage 1 (N = 142)**	**KDIGO stage 2 (N = 60)**	**KDIGO stage 3 (N = 134)**	
**Preoperative continuous**	***Mean (SD)***	***Mean (SD)***	***Mean (SD)***	***Mean (SD)***	
Age (years)	66.75 (11.01)	69.40^*^ (10.96)	70.27 (10.30)	69.81^*^ (12.43)	
BMI	29.74 (7.97)	30.98 (5.87)	30.54 (5.61)	30.23 (5.35)	
Preoperative creatinine (μmol/L)	95.48 (32.16)	101.25 (36.40)	95.28 (20.19)	152.07 (121.10)^*,^&^,#^	
**Preoperative categorical**	***N (%)***	***N (%)***	***N (%)***	***N (%)***	***p-value***
Gender					0.9
*Female (n = 585)*	494 (24.9%)	39 (27.5%)	17 (28.3%)	35 (26.1%)	
*Male (n = 1731)*	1486 (75.1%)	103 (72.5%)	43 (71.7%)	99 (73.9%)	
Preoperative intra-aortic balloon pump					0.02
*Yes (n = 27)*	18 (0.9%)	3 (2.1%)	1 (1.7%)	5 (3.7%)	
*No (n = 2289)*	1962 (99.1%)	139 (97.9%)	59 (98.3%)	129 (96.3%)	
Congestive heart failure					<0.001
*Yes (n = 256)*	177 (8.9%)	23 (16.2%)	15 (25%)	41 (30.6%)	
*No (n = 2060)*	1803 (91.1%)	119 (83.8%)	45 (75%)	93 (69.4%)	
Ejection fraction <35%					0.8
*Yes (n = 224)*	197 (9.9%)	11 (7.7%)	5 (8.3%)	11 (8.2%)	
*sNo (n = 2092)*	1783 (90.1%)	131 (92.3%)	55 (91.7%)	123 (91.8%)	
Type of surgery					<0.001
*CABG only (n = 1509)*	1344 (67.9%)	77 (54.2%)	25 (41.7%)	63 (47.0%)	
*Valve only (n = 251)*	220 (11.1%)	11 (7.7%)	8 (13.3%)	12 (9.0%)	
*CABG + valve (n = 468)*	341 (17.2%)	48 (33.8%)	24 (40.0%)	55 (41.0%)	
*Other cardiac surgery(n = 88)*	75 (3.8%)	6 (4.2%)	3 (5.0%)	4 (3.0%)	
Previous cardiac surgery					<0.001
*Yes (n = 178)*	131 (6.6%)	21 (14.8%)	8 (13.3%)	18 (13.4%)	
*No (n = 2138)*	1849 (93.4%)	121 (85.2%)	52 (86.7%)	116 (86.6%)	
Diabetes mellitus^					0.01
*Type 1 (n = 22)*	18 (0.9%)	2 (1.4%)	0	2 (1.5%)	
*Type 2 (n = 698)*	569 (28.7%)	50 (35.2%)	28 (46.7%)	51 (38.1%)	
*None (n = 1596)*	1393 (70.4%)	90 (63.4%)	32 (53.3%)	81 (60.4%)	
Chronic obstructive pulmonary disease					0.2
*Yes (n = 114)*	90 (4.5%)	10 (7.0%)	4 (6.7%)	10 (7.5%)	
*No (n = 2202)*	1890 (95.5%)	132 (93.0%)	56 (93.3%)	124 (92.5%)	
*Total*	*100%*	*100%*	*100%*	*100%*	
**Perioperative continuous**	***Mean (SD)***	***Mean (SD)***	***Mean (SD)***	***Mean (SD)***	
Cardiopulmonary bypass time (entry to exit minutes)	279.79 (73.91)	314.45^*^ (93.31)	316.45^*^ (92.60)	340.02^*,^& (109.14)	
Clamp time (minutes)	94.77 (36.63)	111.87^*^ (40.65)	114.72^*^ (48.14)	126.36^*,^& (55.70)	
Pump time (minutes)	112.55 (47.95)	135.55^*^ (51.30)	141.98^*^ (66.01)	162.98^*,^& (78.01)	
Number of bypass grafts	5.53 (1.87)	5.44 (2.07)	5.25 (2.49)	5.35 (2.21)	

*Abbreviations*: *AKI* acute kidney injury, *BMI* body mass index, *CABG* coronary artery bypass graft, *KDIGO*, kidney disease: improving global outcomes, *SD* standard deviation.

Note: Conversion factors for units: SCr in to μmol/L to mg/dL x0.0113.

^*^significantly different from No AKI (p < 0.05, Bonferonni).

&significant different from Stage 1 (p < 0.05, Bonferonni).

^#^significantly different from Stage 2 (p < 0.05, Bonferonni).

^p-value with type 1 diabetes only: p = 0.7.

**Table 2:** **Patient outcomes after cardiac surgery**

	**No AKI (N = 1980)**	**KDIGO stage 1 (N = 142)**	**KDIGO stage 2 (N = 60)**	**KDIGO stage 3 (N = 134)**	
	***N (%)***	***N (%)***	***N (%)***	***N (%)***	***p-value***
**Type of dialysis**	N/A	N/A	N/A		N/A
*Continuous Renal Replacement Therapy*				45 (36%)	
*In-Patient Hemodialysis*				75 (60%)	
*Peritoneal Dialysis*				1 (0.8%)	
*Unknown*				4 (3.2%)	
**Discharge destination**					
*Died (n = 148)^*	50 (2.5%)	20 (14.1%)	14 (23.3%)	64 (47.8%)	
*Home (n = 1327)*	1220 (61.6%)	66 (46.5%)	16 (26.7%)	25 (18.7%)	
*Support services (n = 598)*	537 (27.1%)	32 (22.5%)	10 (16.7%)	19 (14.2%)	
*Inpatient facility (n = 204)*	147 (7.4%)	20 (14.1%)	17 (28.3%)	20 (14.9%)	
*Long-term care facility (n = 31)*	19 (1.0%)	4 (2.8%)	3 (5.0%)	5 (3.7%)	
*Other (n = 8)*	7 (0.4%)	0	0	1 (0.7%)	
	***Mean (SD)***	***Mean (SD)***	***Mean (SD)***	***Mean (SD)***	***p-value***
**Length of stay (days)**	14.02 (11.55)	23.83^*^ (35.80)	24.97^*^ (18.31)	32.96^*,^& (33.05)	<0.001

Abbreviations: *AKI* acute kidney injury, *KDIGO* kidney disease: improving global outcomes, *SD* standard deviation.

**^**Mortality only: Χ^2^ (3) = 475.69, *p* < 0.001, Cramer’s V = 0.45.

^*^significantly different from No AKI (p < 0.05, Bonferroni).

&significantly different from Stage 1 (p < 0.05, Bonferroni).

### Cleveland clinic scoring tool

There was a significant overall difference between the KDIGO Stages in the modified Cleveland Clinic score; *p* < 0.001). The mean scores were significantly different between all groups except Stage 1 vs. Stage 2 (*p* < 0.001; Table 3). Like Thakar et al. and Englberger et al. [6,16], we grouped the Cleveland score into four risk categories: low risk (0–2), intermediate risk (3–5), high risk (6–8) and very high risk (≥9). The frequency of patients in each risk category is presented in Table 3. There was a significant relationship between Cleveland Clinic Risk Score Category and AKI Stage with a greater percentage of patients in higher AKI Stages for those classified as higher risk of AKI on the Cleveland tool.

**Table 3:** **Relationship between KDIGO stage and risk categories of the Cleveland tool**

	**No AKI (N = 1980)**	**KDIGO stage 1 (N = 142)**	**KDIGO stage 2 (N = 60)**	**KDIGO stage 3 (N = 134)**
**Overall mean (SD) risk score**	1.61 (1.59)	2.34^*^ (1.68)	2.35^*^ (1.66)	3.69^*,^& (2.07)
**Cleveland risk category**	***N (%)***	***N (%)***	***N (%)***	***N (%)***
**Low risk (0–2; n = 1659)**	1503 (90.6%)	82 (4.9%)	32 (1.9%)	42 (2.5%)
**Intermediate risk (3–5; n = 576)**	429 (74.4%)	55 (9.5%)	25 (4.3%)	68 (11.8%)
**High risk (6–8; n = 75)**	47 (59.5%)	5 (6.3%)	3 (3.8%)	24 (30.4%)
**Very high risk (≥9; n = 1)**	1 (100%)	0	0	0

Abbreviations: *AKI* acute kidney injury, *KDIGO* kidney disease: improving global outcomes, *SD* standard deviation.

^*^significantly different from No AKI (p < 0.05, Bonferonni).

&significantly different from Stage 1 (p < 0.05, Bonferonni).

**Figure 1:**
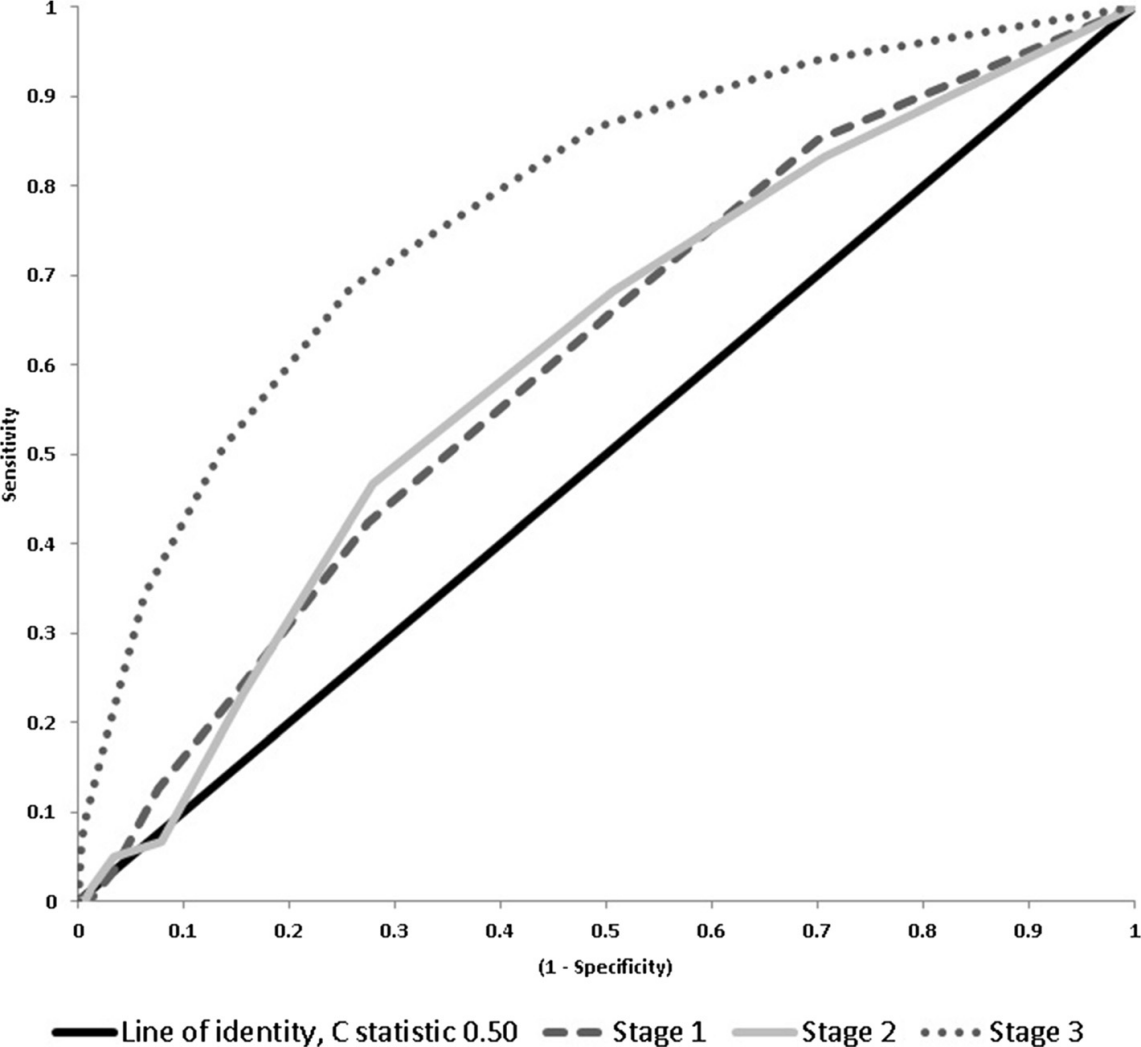
**Area under the curve for Cleveland score for patients with AKI stage 1 (Grey oval dash), stage 2 (Grey solid) and stage 3 (Grey circle dotted).** The black line represents the line of identity, c statistic 0.50. Abbreviations: KDIGO, Kidney Disease: Improving Global Outcomes.

To test the validation of the Cleveland Clinic score in our population, we generated receiver operating characteristics curves for predicting AKI Stages 1, 2, and 3 using the Cleveland Clinic score. The area under the curve (AUC) for the Cleveland score was 0.61 (95% CI: 0.56 to 0.65; *p* < 0.001) for Stage 1, 0.61 (95% CI: 0.54 to 0.68; *p* < 0.01) for Stage 2, and 0.78 (95% CI: 0.74 to 0.82; *p* < 0.001) for Stage 3 (Figure 1). We also were interested in whether combining Stage 1 and Stage 2 would improve the AUC of the model; however, the value remained the same (0.61, 95% CI: 0.57 to 0.65; *p* < 0.001).

We did a post-hoc analysis of calibration and found the model to have good calibration for prediction of Stage 3 (*p* = 0.84), moderate calibration for Stage 1 (*p* = 0.29), and poor discrimination for Stage 2 (*p* = 0.08), with higher p-values indicating better calibration. However, given that calibration is very sensitive to sample size, the results should be interpreted with caution.

## Discussion

We used the Cleveland Clinic scoring tool to predict different stages of AKI within 5 days of cardiac surgery in patients treated between 2007 and 2011 in the RQHR. Like previous studies [6,27-29], we found that the Cleveland Clinic tool performed well in discriminating patients who required dialysis (Stage 3) from patients without any kidney injury (no AKI). However, the score exhibited much weaker discrimination for less severe AKI (Stages 1 and 2). To our knowledge, the present study is the first study to evaluate the Cleveland Clinic score for less severe AKI in a Canadian cohort.

We found that the AUC for the Cleveland Clinic score for patients requiring dialysis was 0.78, which is marginally lower than the values seen in previous studies with values consistently >0.80 [6,27-29]. A previous study in Germany [33] found that the Cleveland tool had an AUC of 0.66, but variable definitions for risk factors and limitations in their risk factor data may have resulted in the low AUC value. To our knowledge, two previous studies have used the Cleveland Clinic score in AKI patients not requiring dialysis [6,27]. Englberger et al. [6] defined severe AKI as an increase in SCr to >2.0 mg/dL (>176.8 μmol/L) and a 2-fold increase compared to baseline, whereas Kiers et al. used the RIFLE criteria to identify patients with ≥50% relative increase in SCr compared to baseline [27]. In both studies, the Cleveland Clinic score still had the highest AUC compared to other scoring tools, but the AUC for AKI not requiring dialysis was lower than the AUC for AKI-D [6,27]. We found that the Cleveland Clinic score had lower discriminative power for AKI Stages 1 and 2 (0.61 for both) compared to these previous studies.

Several factors may have contributed to the lower AUCs in our cohort. We hypothesize that the difference in AUC is likely due to true differences in the study population and center-specific practices, rather than limitations in the definitions of the predictors and outcomes. Our patients may have been in better preoperative health than other studies, whose patients had more comorbidities and poorer preoperative cardiac function [6,16,27,28], similar to the patients in the original Cleveland Clinic score development study [16]. The Cleveland Clinic score was also validated in a cohort with a higher proportion of non-isolated CABG surgeries [16]. Isolated CABG surgery has a lower incidence of AKI compared to valve and combined surgeries [4,7], and this is reflected in the Cleveland Clinic score as non-isolated CABG surgeries are assigned higher point values [16]. The combination of healthier patients in our cohort and less complex surgeries than other cohorts [6,28,33] may have resulted in lower discriminative power. The absence of data on whether the surgery was emergent or not, one of the stronger contributors to the Cleveland Clinic scoring tool [16], may also have reduced the predictive power of the Cleveland score. The way emergent surgery is recorded would also be important as the outcomes of patients labeled as “emergent” can be vastly different.

Unlike the original Cleveland Clinic score paper [16], female gender, COPD, type 1 diabetes and LVEF <35% were not significant predictors of AKI Stage in our cohort. As noted above, better preoperative health of our cohort may be a contributing factor. In addition, there was variation in the way LVEF was measured and recorded among clinicians at our center. Typically patients with LVEF <20% are not operated on at our center. Approximately half the LVEF data were coded as binary (<35%, ≥35%) so we were unable to examine the distribution of patients with LVEF levels and determine if our population had more LVEF closer to 35% compared to other studies. Therefore, selection bias may have contributed to the lack of LVEF effect in our study. We had a very low proportion of patients with type 1 diabetes and the lack of information on insulin use likely underestimated the sample size for that predictor and reduced our power. Also, Thakar [17] defined COPD as a history of COPD requiring medications; we were not able to determine from the records whether patients were medicated or not. Moreover, COPD is used inconsistently as a general descriptor of patients with breathing difficulties or a history of smoking without requiring medical tests prior to diagnosis. Thus, it is possible that our patients had a lower severity of COPD than other cohorts.

Almost half of all patients who progressed to Stage 3 AKI after cardiac surgery died, whereas 23.3% died in Stage 2 and 14.1% in Stage 1. Therefore, even AKI Stages 1 and 2 are associated with a high degree of mortality. Even though the numbers were small, 2.8% of patients in Stage 1 and 5.0% in Stage 2 required long-term care facilities. Patients in Stage 1 and 2 also stayed significantly longer in hospital than patients with no AKI. Therefore, our data suggest that even early stages of AKI are associated with considerable burden on the patient and the health care system, warranting a need for preoperative risk assessment. These results were similar to other studies, and further illustrate the need for prediction models that are accurate in predicting earlier stages of AKI.

Ideally, a preoperative risk stratification tool would identify high-risk patients so that prevention strategies could be started earlier, which would improve quality of care, patient management, and resource allocation. These measures may include: avoiding nephrotoxic medications whenever possible, optimizing fluid volume and perfusion pressure, correcting electrolyte imbalances, monitoring indicators of renal function such as SCr and urine output, treating infection and oliguria quickly, preventing hyperglycemia, and considering alternatives to therapies that may result in an elevated risk of kidney injury, such as those involving traditional contrast agents [3,5,32,34,35]. Although several biomarkers have shown promise in detecting AKI at 24 hours after surgery, intraoperative or immediately postoperative detection remains elusive [1].

## Conclusions

We have demonstrated the validity of the Cleveland Clinic score for predicting risk of severe AKI (Stage 3) in a large, diverse sample of Canadian patients undergoing cardiac surgery. The score was less successful in stratifying patients into Stages 1 or 2. However, these earlier and milder forms of AKI were still associated with outcomes that have a considerable impact on health care expenditure, such as increased time in hospital, home care and other support services, and time in rehabilitation step-down units. AKI also greatly increases the risk of chronic kidney disease and downstream end-stage renal disease. Therefore, future research is needed to create alternative risk scores that can accurately predict earlier stages of AKI in order to develop interventions that may prevent these adverse events.

## Additional file

Additional file 1:
**Modified Cleveland Clinic score.**
