# Supplementary material for: Validating a scoring tool to predict acute kidney injury (AKI) following cardiac surgery
Source: Can J Kidney Health Dis. 2015 Jan 30;2:3. doi: 10.1186/s40697-015-0037-x (PMC4349478; doi:10.1186/s40697-015-0037-x)
Supplement: Additional file 1: — Modified Cleveland Clinic score. [file 40697_2015_37_MOESM1_ESM.docx]

**Appendix 1. Modified Cleveland Clinic score.**

| **Risk Factor** | **Points** |
| --- | --- |
| **Female Gender** | 1 |
| **Congestive Heart Failure** | 1 |
| **Left ventricular ejection fraction <35%** | 1 |
| **Preoperative use of IABP** | 2 |
| **COPD** | 1 |
| **Type 1 diabetes** | 1 |
| **Previous cardiac surgery** | 1 |
| **Emergency Surgery (not included)** | (2) |
| **Surgery Type**  ***Valve only***  ***CABG + Valve***  ***Other cardiac surgeries*** | 1  2  2 |
| **Preoperative creatinine**  ***106.1µmol/L to 185.64 µmol/L***  ***>185.64 µmolL*** | 2  5 |

Abbreviations: CABG, coronary artery bypass graft; COPD, chronic obstructive pulmonary disease; IABP, intra-aortic balloon pump

**minimum score is 0, maximum score is 15*
